# Automated Assessment to Predict Lethal Arrhythmias in Brugada Syndrome: Significance of R' in Lead III

**DOI:** 10.1002/joa3.70166

**Published:** 2025-08-25

**Authors:** Daiki Shako, Satoshi Nagase, Naoya Kataoka, Toshihiro Nakamura, Satoshi Oka, Yuichiro Miyazaki, Akinori Wakamiya, Kenzaburo Nakajima, Nobuhiko Ueda, Tsukasa Kamakura, Mitsuru Wada, Kohei Ishibashi, Yuko Inoue, Koji Miyamoto, Takeshi Aiba, Kengo Kusano

**Affiliations:** ^1^ Department of Cardiovascular Medicine National Cerebral and Cardiovascular Center Suita Japan; ^2^ Department of Cardiovascular Medicine, Graduate School of Medical Science Kyoto Prefectural University of Medicine Kyoto Japan; ^3^ Department of General Internal Medicine 3 Kawasaki Medical School General Medical Center Okayama Japan; ^4^ Second Department of Internal Medicine University of Toyama Toyama Japan; ^5^ Department of Advanced Arrhythmia and Translational Medical Science National Cerebral and Cardiovascular Center Suita Japan

**Keywords:** Brugada syndrome, electrocardiogram, epicardial mapping, fragmentation, sudden cardiac arrest, ventricular fibrillation

## Abstract

**Background:**

Previous investigations of predictors of sudden cardiac arrest (SCA) in Brugada syndrome (BrS) have been conducted using manual electrocardiogram (ECG) measurement, which lacks objectivity. This study aimed to examine predictive factors for SCA in BrS using automated ECG measurements.

**Methods:**

In 270 patients with BrS, ECGs were recorded using the same electrocardiograph when a Type 1 ECG was initially observed. The data were digitally acquired and analyzed using identical software. Right precordial leads in the upper one and two intercostal spaces were recorded in all patients.

**Results:**

During a median follow‐up of 88 months, 28 patients (10%) experienced SCA. Multivariate analysis showed that a history of SCA, history of syncope, R' duration ≥ 18 ms in lead III, and maximum corrected Tpeak‐end interval ≥ 137 ms in right precordial leads were independent predictors of SCA. To simplify clinical verification, the presence of an R' wave in lead III was used as a cut‐off surrogate. Visual evaluation confirmed that this R' wave remained an independent predictor of SCA in multivariate analysis. All seven patients with BrS who underwent epicardial mapping had fragmented potential at the base of the right ventricular inferior/inferolateral wall. This area corresponded to the most inferior and right‐sided region of the ventricle, suggesting lead III may best reflect this region.

**Conclusion:**

Automated ECG measurement revealed that an R' duration ≥ 18 ms in lead III predicted SCA in patients with BrS. Visual assessment also identified the R' wave in lead III as a novel predictor of SCA.

## Graphical abstract

Fragmented potentials in the inferior/inferolateral wall of the right ventricle might reflect the R' wave in lead III, indicating the presence of extensive arrhythmic substrate in patients with Brugada Syndrome.

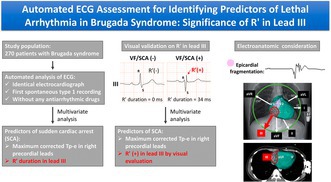

## 1 Introduction

Brugada syndrome (BrS) is characterized by coved‐type ST‐segment elevation in the right precordial leads, with a higher risk of sudden cardiac arrest (SCA) owing to polymorphic ventricular tachycardia or ventricular fibrillation (VF) [1]. Although various treatment strategies, such as implantable cardiovascular defibrillators and epicardial ablation for the prevention of VF/SCA, have proven effective, robust predictors of VF/SCA remain a matter of debate.

Predictors of VF/SCA in BrS based on electrocardiogram (ECG) findings have been reported in numerous publications; however, the results have been inconsistent [2, 3, 4, 5, 6, 7, 8, 9, 10, 11, 12, 13, 14, 15, 16]. Some of the reasons include incorrect manual measurements by the analysts and the difficulty of proving reproducibility owing to the complicated measurement method. In this regard, it is crucial to reevaluate predictors and search for new ones using automated measurement, with high objectivity and reproducibility. Studies on P waves using automated ECG measurement have been previously reported, including the software utilized in the present study [17, 18].

Meanwhile, the use of artificial intelligence to identify risk factors is also gaining attention. However, a major problem with artificial intelligence studies is the uncertainty of causal relationships; this raises concerns about the overestimation of risk and makes it difficult to identify pathophysiology and the mechanism of BrS.

Therefore, this study aimed to investigate the risk factors for the occurrence of VF/SCA by evaluating various ECG markers for BrS by using automated ECG measurement. Additionally, the novel findings obtained from the automated analysis were revalidated using manual measurements.

## 2 Methods

### 2.1 Patient Population and Clinical Data

This study was approved by the Institutional Research Board of the National Cerebral and Cardiovascular Center (M22‐031‐13 and M26‐148‐16). The need for informed consent was waived by disclosing information about the study to the public and ensuring that study subjects had an opportunity to refuse to participate. This study was conducted according to the principles of the Declaration of Helsinki.

BrS was defined as the manifestation of type 1 ECG pattern, characterized by a coved‐type ST‐segment elevation ≥ 0.2 mV, followed by a negative T wave in leads V1 or V2 at the second, third, or fourth intercostal space, with or without the administration of a class IC antiarrhythmic drug (pilsicainide) [19].

At our institution, 378 out of 470 patients with BrS who met the diagnostic criteria had a spontaneous type 1 ECG pattern. Among these, we analyzed the first ECG that simultaneously met the following criteria: leads V1–V3 were recorded over one and two higher intercostal spaces in addition to the standard position; a type 1 ECG pattern was observed; and the recording was performed using the Fukuda Denshi system (Fukuda Denshi Co, Tokyo, Japan) implemented after April 2010. Patients on antiarrhythmic drugs were excluded, and the final investigation was conducted in 270 patients.

Clinical data, including age, sex, family history of sudden cardiac death (in those aged < 45 years), history of syncope episodes, history of VF episodes, and VF inducibility with programmed ventricular pacing, were obtained from patient records. The follow‐up period was defined as the time from the initial medical examination at our institute until VF/SCA or the last in‐person or telephone visit.

### 2.2 ECG Recording and Analysis

All ECGs were recorded, digitally acquired, and analyzed using an identical electrocardiographic system with attached software (Fukuda Denshi Co) [17, 20]. In addition to the standard 12 leads, leads V1–V3 on the upper one and two intercostal spaces were analyzed in all patients. Consequently, the nine right precordial leads of V1–V3 on the standard and upper intercostal spaces were evaluated. The numerical values of the various parameters (RR, PQ, QRS, QT, and Tpeak‐end [Tpe] intervals; amplitude and duration of R, S, R', and J waves in all leads; maximum QT interval; and maximum Tpe interval among all leads and nine right precordial leads) on ECG were analyzed using the automatic ECG analysis software (Fukuda Denshi TG02‐06). The QT and Tpe intervals, corrected for heart rate by Bazett's formula, were named QTc and cTpe, respectively, and were studied similarly. In the automated analysis, a J wave was defined as a notch of ≥ 0.1 mV on the downslope of the R wave, in cases where no S wave was present. As usual, an R' wave was defined, in both automated analysis and visual inspection, as an upward deflection occurring after the R wave, characterized by an initial descent below the isoelectric line (i.e., forming an S wave), followed by a subsequent rise exceeding the baseline (> 0 mV). The parameter (R' in lead III) that showed a significant difference after data analysis was evaluated by visual inspection in a blind manner for validity (by D.S. and S.N.).

### 2.3 Electrophysiological Testing

After obtaining written informed consent from patients, an electrophysiological study was conducted in 89 patients (32%), as described previously [21]. The criterion for the induction of ventricular arrhythmia was VF or polymorphic ventricular tachycardia lasting ≥ 30 s or requiring direct current shock induced at a coupling interval of ≥ 200 ms with a maximum of two extrastimuli.

### 2.4 Genetic Testing for *SCN5A*

Genetic testing to detect pathogenic variants in *SCN5A* was performed in 76 patients (28%) with BrS, as previously described [22].

### 2.5 Epicardial Fragmentation Mapping

To investigate the characteristics of lead III, we examined the location of fragmented potentials with bipolar recording in the epicardium of seven male patients with BrS (aged 42 ± 8 years) who underwent right ventricular epicardial mapping and ablation for frequent VF episodes, as described previously [23]. Three patients were included in this study on automated ECG analysis, and two underwent epicardial mapping in another institution (University of Toyama). Under general anesthesia, an epicardial approach with mini‐thoracotomy was utilized for mapping and ablation in a three‐dimensional mapping system (Carto 3, Biosense Webster, Irvine, CA, USA). Epicardial substrate maps were obtained using a multipolar mapping catheter (DECANAV, Biosense Webster). We recorded local bipolar electrograms with a 30‐ to 250‐Hz bandwidth using a digital recording system (LabSystem PRO, Bard Electrophysiology, Lowell, MA, USA or RMC‐5000, Nihon Kohden, Tokyo, Japan). Fragmented potential was defined as the intra‐ or post‐QRS sharp potential with multiple deflections.

### 2.6 Statistical Analysis

Data were analyzed using EZR (64‐bit; R Core Team, Vienna, Austria). Categorical variables are expressed as frequencies and percentages and analyzed using the Chi‐Squared test [24]; Continuous variables were compared using a nonparametric Mann–Whitney test.

A VF/SCA event was defined as the appropriate implantable cardioverter‐defibrillator shock against VF and aborted cardiac arrest attributable to VF. The receiver‐operating characteristic curve and area under the curve were evaluated to select the optimized cut‐off value for the prediction of VF/SCA during follow‐up. Event analysis over time was performed using the Cox proportional hazards regression model.

Univariate and multivariate analysis included the following covariates: age, sex, history of syncope/VF, *SCN5A* variant, VF inducibility with programmed pacing, and parameters of ECG. Risk was quantified as a hazard ratio with a 95% confidence interval. *p* < 0.05 was considered statistically significant. Because 22 factors of ECG parameters were found to be significant in the univariate analysis, the *p* value cut‐off for the multivariate analysis was set at 0.00135, using the Bonferroni correction.

Finally, after excluding similar factors and prioritizing heart rate‐corrected data, we performed multivariate analysis on the following four ECG parameters: R' duration (or visual R') in lead III, maximum QTc in right precordial leads, cTpe in lead aVL, and maximum cTpe in right precordial leads. Survival curves were plotted by the Kaplan–Meier method and analyzed using the log‐rank test.

## 3 Results

The baseline characteristics of the study population are summarized in Table 1. The mean age of the patients was 43 ± 13 years, and 258 (96%) were male. A history of VF episodes was reported in 18 patients (7%), syncope in 57 (21%), while 195 patients (72%) reported no symptoms. Gene analysis was performed in 76 patients and showed that an *SCN5A* gene variant was present in 18 (24%).

**TABLE 1:** Baseline characteristics.

Characteristic	Value
Age, years	44 ± 13
Male, sex	258 (96%)
Symptom	
VF	18 (7%)
Syncope	57 (21%)
Asymptomatic	195 (72%)
FH of SCD	56 (21%)
Induced VF with PES (performed: *n* = 89)	20 (23%)
*SCN5A* variant (performed: *n* = 76)	18 (24%)
ICD implantation	73 (27%)

Abbreviations: FH of SCD, family history of sudden cardiac death; ICD, implantable cardioverter defibrillator; PES, programmed electrical stimulation; VF, ventricular fibrillation.

In the electrophysiological study, VF was induced in 20 out of 89 patients (22%). An implantable cardioverter‐defibrillator was used in 73 patients (27%). During a median follow‐up of 88 (interquartile range: 25–173) months, 28 patients (10%) experienced VF/SCA. The relationship between the occurrence of events over the entire follow‐up period and the factors examined is shown in Tables S1 (clinical parameter) and S2 (ECG parameter).

**FIGURE 1:**
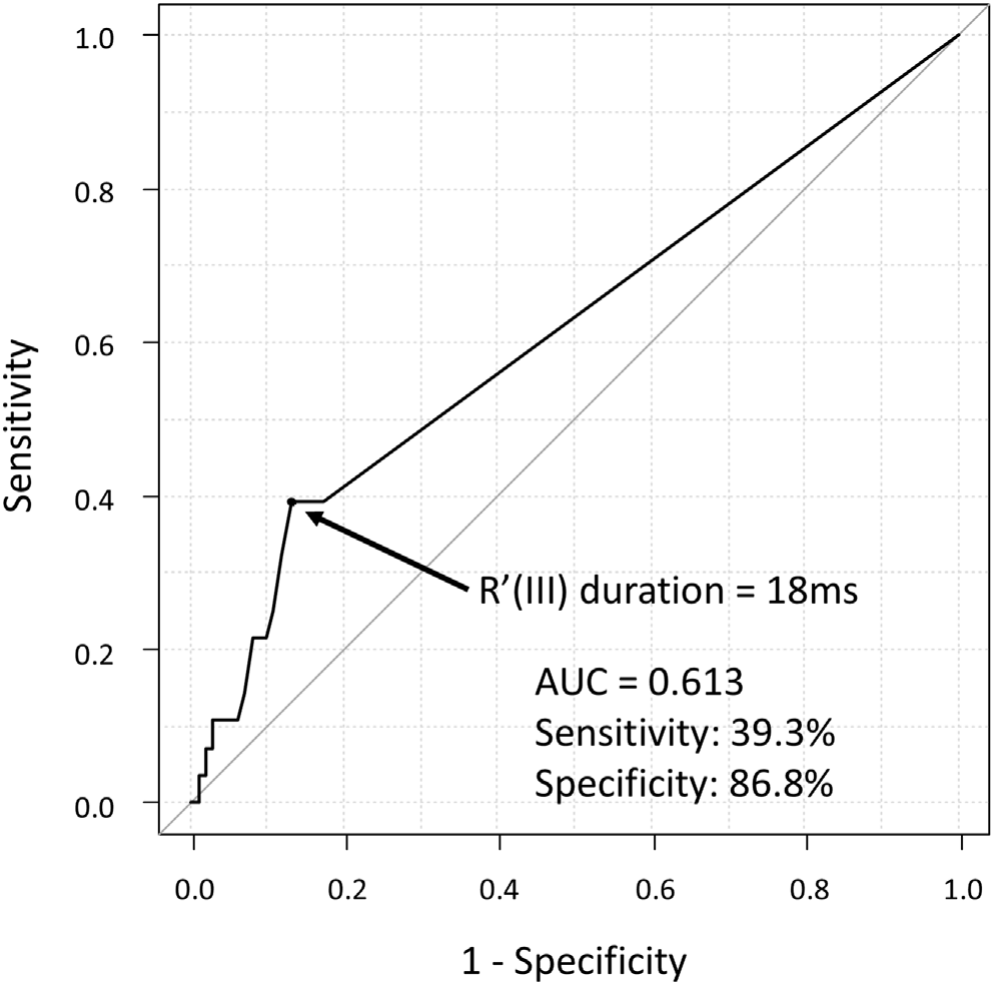
Receiver‐operating characteristic curve for R' wave duration in lead III. AUC, area under the curve; R' (III), R' wave in lead III.

**FIGURE 2:**
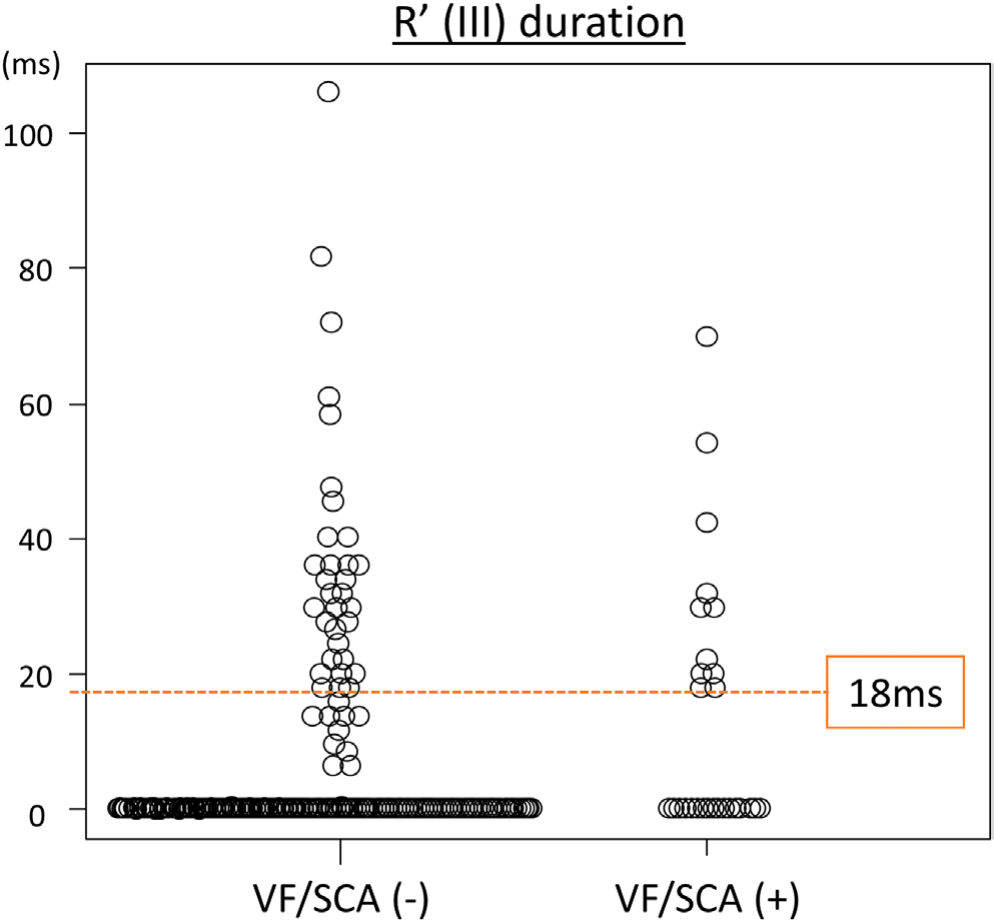
Comparison of R' wave duration in lead III. R' (III), R' wave in lead III; SCA, sudden cardiac arrest; VF, ventricular fibrillation; VF/SCA, appropriate implantable cardioverter‐defibrillator shock against VF and aborted cardiac arrest attributable to VF.

Cut‐off values for significantly different factors were determined using the receiver operating characteristic curve analysis, and univariate data from Cox analyses performed on the basis of these are presented in Table S3. Receiver operating characteristic curve analysis for the R' wave duration in lead III revealed that the optimized cut‐off point in the occurrence of VF/SCA during follow‐up was 18 ms (area under the curve = 0.613; Figure 1). Bonferroni correction showed that maximum QTc ≥ 481 ms in the nine right precordial leads (*p* < 0.001), R' duration ≥ 18 ms in lead III (*p* < 0.001), Tpe ≥ 93 ms in lead aVL (*p* < 0.001), cTpe ≥ 98 ms in lead aVL (*p* < 0.001), maximum cTpe ≥ 137 ms in the nine right precordial leads (*p* < 0.001), and maximum cTpe ≥ 137 ms in all leads (*p* = 0.001) were associated with VF/SCA. Multivariate analysis showed that a history of VF, history of syncope, R' duration ≥ 18 ms in lead III, and maximum cTpe ≥ 137 ms in the nine right precordial leads were independent predictors of VF/SCA (Table 2). Figure 2 shows the relationship between the R' duration in lead III and VF/SCA.

**TABLE 2:** Predictive factors of VF/SCA (1).

	Univariate analysis			Multivariate analysis		
	HR	95% CI	*p*	HR	95% CI	*p*
Sex: male	1.25	0.17–9.19	0.828			
FH of SCD	0.81	0.30–2.16	0.677			
*SCN5A* variant	1.20	0.43–3.36	0.732			
VF induced in EPS	0.51	0.11–2.23	0.369			
History of VF	10.66	4.95–22.99	< 0.001	19.73	6.52–59.75	< 0.001
History of syncope	2.44	1.15–5.18	0.020	5.26	1.81–15.27	0.002
QTc max duration (V1–3; Normal–upper 2ICS) ≥ 481 ms	4.64	2.14–10.08	< 0.001	2.10	0.82–5.41	0.123
**R' (III) wave duration ≥ 18 ms**	**3.68**	1.71–7.93	**< 0.001**	**2.54**	1.07–6.04	**0.034**
cTp‐e (aVL) ≥ 93 ms	3.80	1.79–8.03	< 0.001	2.13	0.95–4.80	0.067
cTp‐e max duration (V1–3; Normal‐upper 2ICS) ≥ 137 ms	3.84	1.73–8.53	< 0.001	2.96	1.17–7.47	0.022

Abbreviations: CI, confidence interval; cTp‐e, Tp‐e intervals corrected for heart rate by Bazett's formula; EPS, electrophysiologic study; FH of SCD, family history of sudden cardiac death; HR, hazard ratio; ICS, intercostal space; QTc, QT corrected for heart rate by Bazett's formula; SCA, sudden cardiac arrest; Tp‐e, Tpeak‐end; VF, ventricular fibrillation.

**FIGURE 3:**
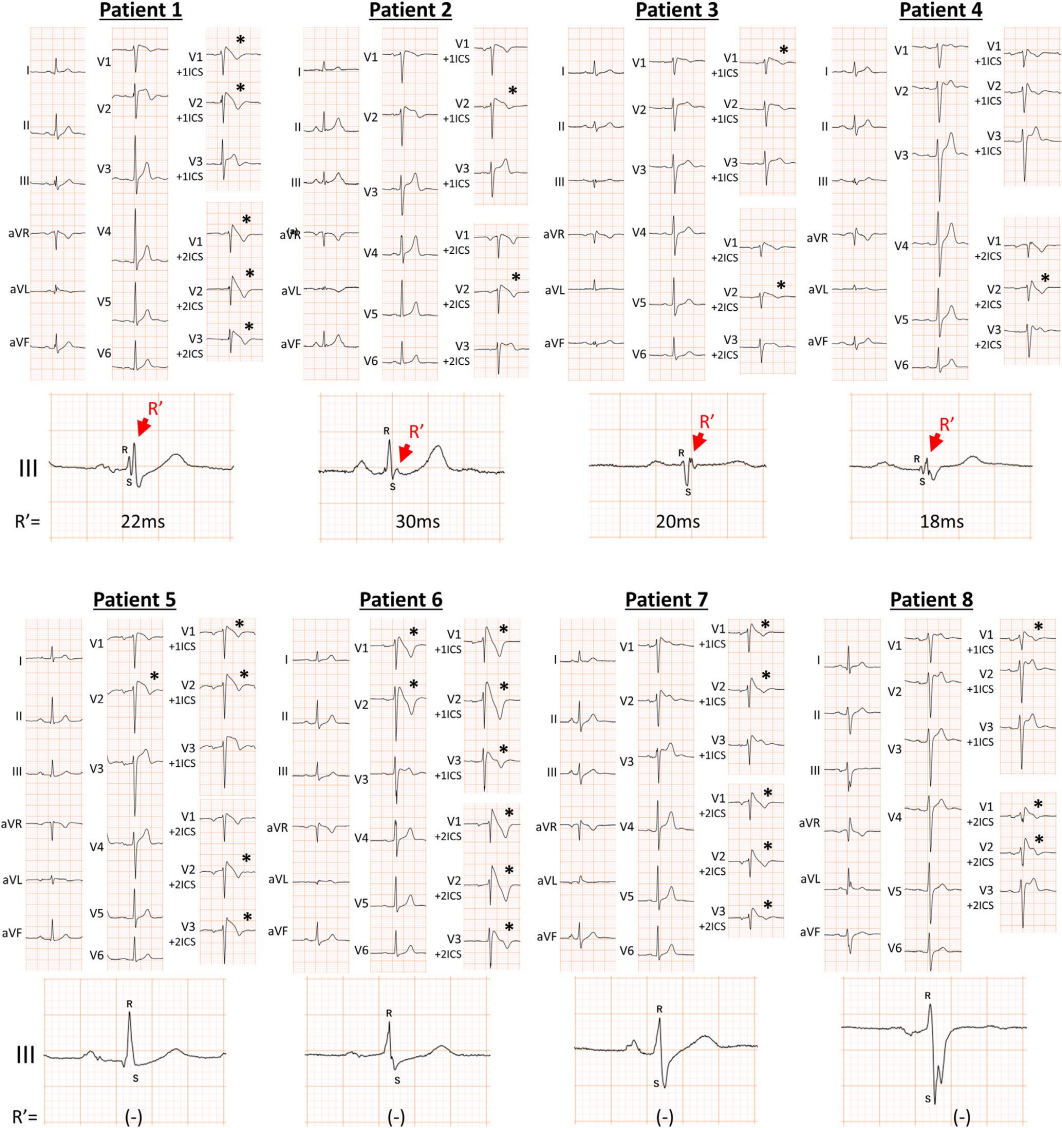
Representative electrocardiograms (ECGs) with and without events. During the follow‐up period, ventricular fibrillation/sudden cardiac arrest is observed in patients 1–4 but not in patients 5–8. R' in lead III is observed in patients 1–4. The values in the bottom row in patients 1–4 represent the duration of the R' wave in lead III, calculated using automated analysis. Red arrows indicate R' in lead III. Asterisks indicate type 1 ECG. Patients 1, 2, and 4 do not meet the definition of fragmented QRS in the inferior leads (≥ 3 spikes in ≥ 2 leads) as defined by Morita et al. [11].

**FIGURE 4:**
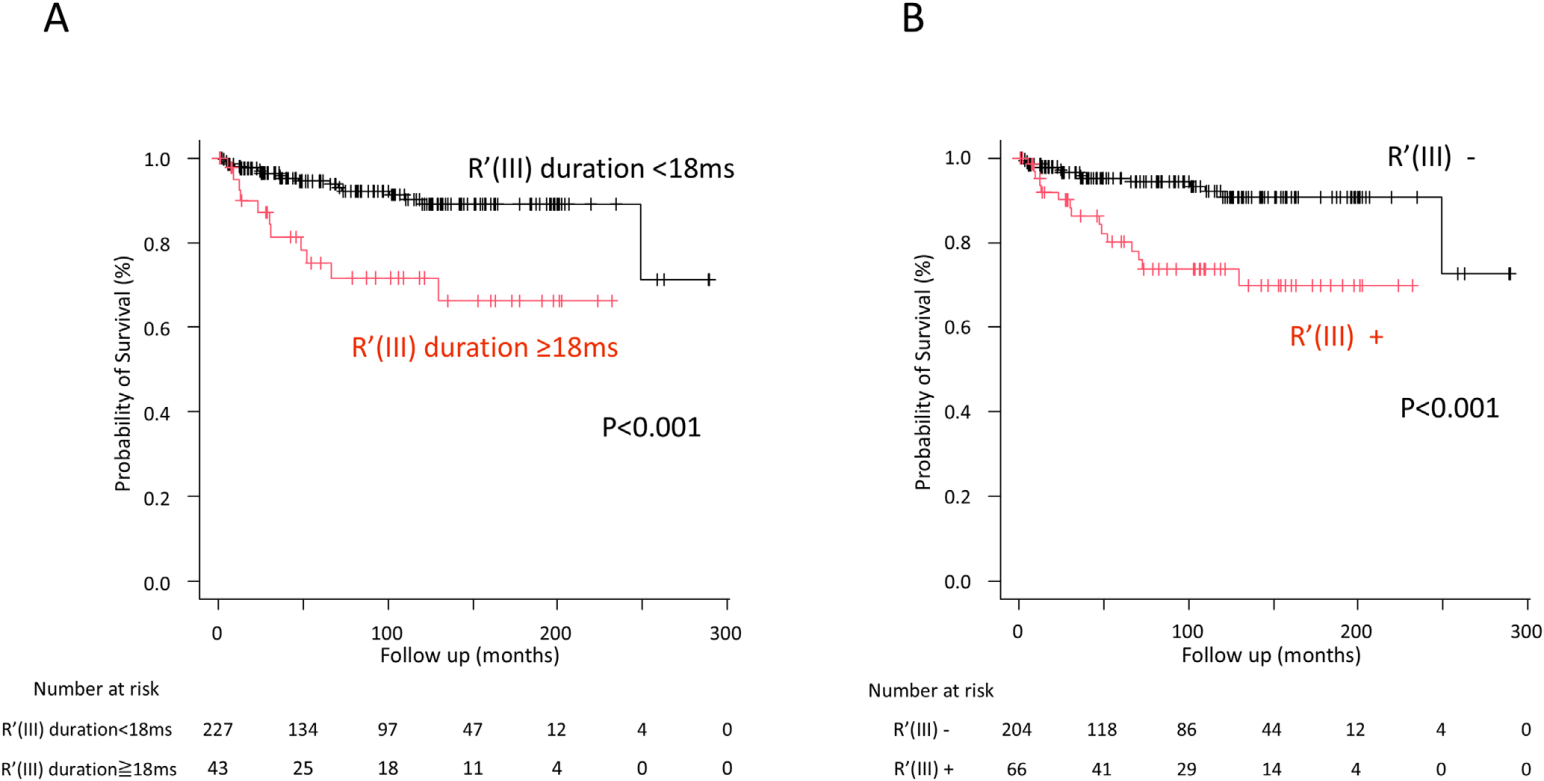
Kaplan–Meier analyses of freedom from ventricular fibrillation/sudden cardiac arrest. (A) Comparison of groups with an R' wave duration in lead III, calculated by automatic analysis, of ≥ 18 ms and < 18 ms. (B) Comparison between the group with a visually identified R' wave in lead III and the group without it. R' (III), R' wave in lead III.

To facilitate clinical validation through manual measurements, the visual presence of the R' wave in lead III was utilized as a surrogate for the cut‐off value. Evaluation using visual inspection confirmed that the presence of the R' wave in lead III remained an independent predictor of VF/SCA in the multivariate analysis (Table 3; Figure 3). Figure 4A,B show Kaplan–Meier analyses of freedom from VF/SCA during follow‐up in patients with or without an R' wave duration ≥ 18 ms in lead III and with or without the presence of an R' wave in lead III through visual evaluation, respectively. The patients with an R' wave duration ≥ 18 ms and the presence of an R' wave by visual evaluation in lead III had a significantly poorer prognosis than the others (*p* < 0.001 and *p* < 0.001, respectively).

**TABLE 3:** Predictive factors of VF/SCA (2).

	Univariate analysis			Multivariate analysis		
	HR	95% CI	*p*	HR	95% CI	*p*
Sex: male	1.25	0.17–9.19	0.828			
FH of SCD	0.81	0.30–2.16	0.677			
*SCN5A* variant	1.20	0.43–3.36	0.732			
VF induced in EPS	0.51	0.11–2.23	0.369			
History of VF	10.66	4.95–22.99	< 0.001	18.96	6.35–56.58	< 0.001
History of syncope	2.44	1.15–5.18	0.020	4.86	1.67–14.18	0.004
QTc max duration (V1–3; Normal‐upper 2ICS) ≥ 481 ms	4.64	2.14–10.08	< 0.001	1.84	0.71–4.77	0.213
**Presence of R' (III) by visual evaluation**	**3.78**	1.76–8.05	**< 0.001**	**3.29**	1.42–7.64	**0.006**
cTp‐e (aVL) ≥ 93 ms	3.80	1.79–8.03	< 0.001	2.49	1.12–5.54	0.025
cTp‐e max duration (V1–3; Normal–upper 2ICS) ≥ 137 ms	3.84	1.73–8.53	< 0.001	3.60	1.39–9.36	0.009

Abbreviations: CI, confidence interval; cTp‐e, Tp‐e intervals corrected for heart rate by Bazett's formula; EPS, electrophysiologic study; FH of SCD, family history of sudden cardiac death; HR, hazard ratio; ICS, intercostal space; QTc, QT corrected for heart rate by Bazett's formula; SCA, sudden cardiac arrest; Tp‐e, Tpeak‐end; VF, ventricular fibrillation.

**FIGURE 5:**
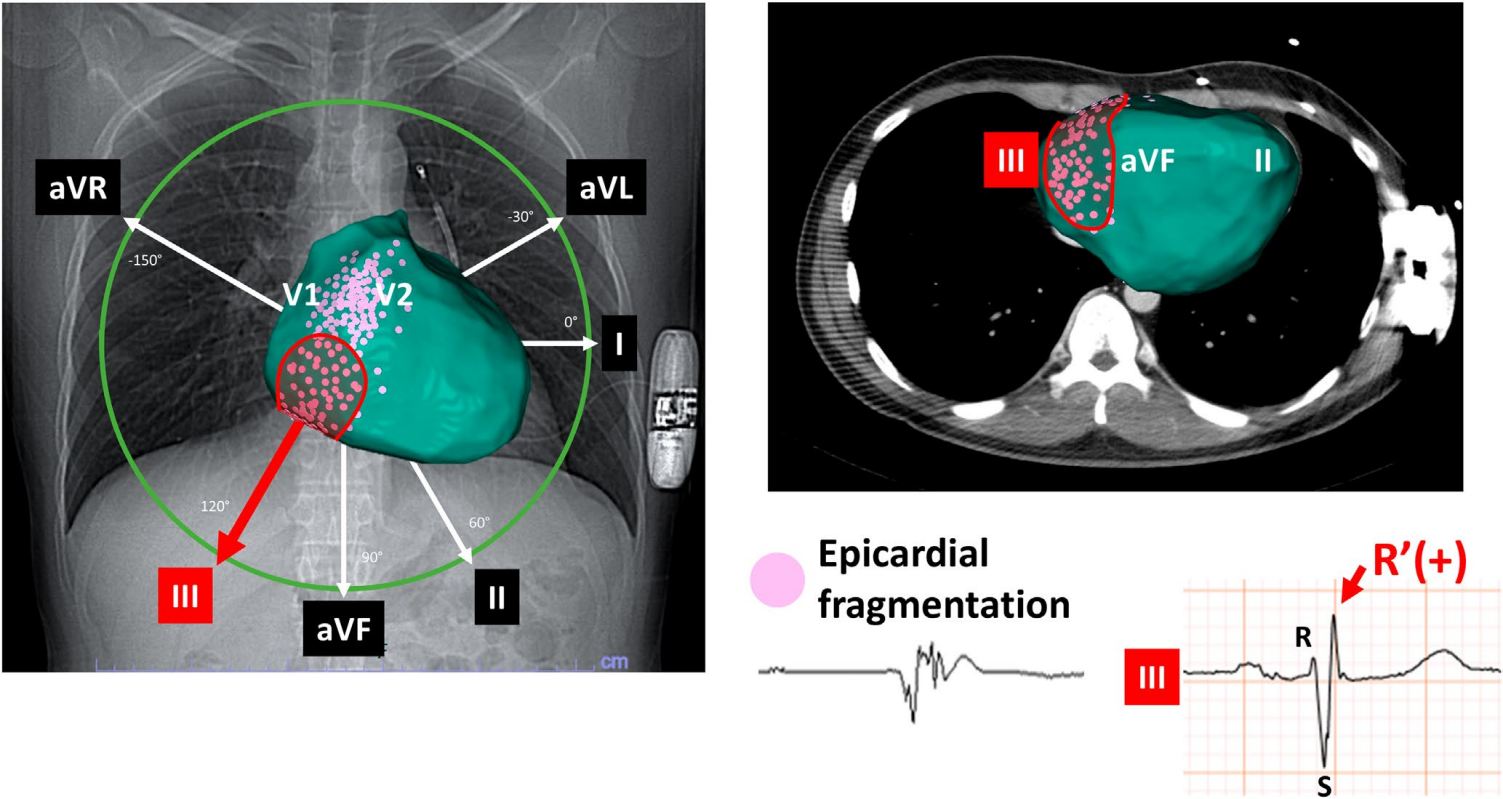
Expected spatial relationship between the recording sites of epicardial fragmented potentials and lead III. In all seven patients with Brugada syndrome who experience recurrent ventricular fibrillation and undergo epicardial mapping, fragmented potentials are recorded in the inferior/inferolateral wall of the right ventricle, where lead III is particularly expected to be preferentially reflected. The pink tags indicate the recording sites of the fragmented potentials.

All the seven patients with BrS who underwent epicardial mapping had fragmented potential at the base of the right ventricular inferior/inferolateral wall. The most inferior and right‐sided region of the entire ventricle corresponded to this area (Figure 5). Thus, it is expected that lead III may reflect this region most preferentially.

## 4 Discussion

The present study elucidated the following points: (1) through highly objective analysis using automated ECG measurements, not only the previously reported maximum cTpe in the right precordial leads but also a newly identified R' ≥ 18 ms in lead III were independent predictors of lethal events in BrS; (2) based on the automated measurement of R' ≥ 18 ms in lead III, the presence of an R' wave in lead III, as determined using visual assessment, was also an independent predictor; and (3) in a small number of patients with BrS with a history of VF, fragmented potentials were recorded in the inferior/inferolateral wall of the right ventricle, corresponding to lead III, during epicardial mapping. This suggests a possible association with the R' wave in lead III. The uniformity of the data, measured with a single software program using a single electrocardiograph at a single institution, can be of great value. The fact that the maximum cTpe in the right precordial leads was significant in this study, similar to previously reported risk factors, suggests the validity of the analytical methods used [2, 12, 13, 14, 25].

Among the 270 patients, lethal events occurred in 25.6% (11/43) of those with an R′ wave ≥ 18 ms in lead III, and in 7.5% (17/227) of those with an R′ wave < 18 ms. Similarly, such events were observed in 22.7% (15/66) of patients with visually identified R′ waves in lead III, and in 6.4% (13/204) of those without them. The sensitivity, specificity, positive predictive value (PPV), and negative predictive value (NPV) for R′ ≥ 18 ms were 39.3%, 86.8%, 25.6%, and 92.5%, respectively. For visual R′ identification, the corresponding values were 53.6%, 78.9%, 22.7%, and 93.6%. These findings indicate that although both markers have modest sensitivity and PPV, their high specificity and NPV suggest they may be useful for identifying patients at low risk.

### 4.1 Relationship Between R' in Lead III and Lethal Cardiac Events

There are several possible mechanisms by which R' in lead III was an independent predictor of cardiac events. First, all patients in this study had spontaneous type 1 ECG, which suggests that at least some arrhythmic substrate is present in the right ventricular outflow tract [26]. Furthermore, the abnormal findings in the inferior leads, especially in lead III, may suggest an involvement of the arrhythmogenic substrate in the inferior right ventricular region. Indeed, although epicardial mapping was performed in only seven patients, fragmented potentials were consistently recorded in the most inferior and right‐sided of the ventricle in those with recurrent VF episodes. It is expected that lead III may reflect this region most preferentially. These findings indicate the existence of a broad substrate of arrhythmogenicity. An R' wave in lead III was observed before ablation in three of the seven patients who underwent epicardial mapping, and it persisted in all of them after ablation. This may be attributed to the limited ablation area and/or the presence of pre‐existing widespread conduction delay.

Regarding the mechanism of R' in lead III, it may first indicate fragmentation of the QRS itself [11] which may signify a severe conduction delay in the inferior wall. In addition, although the early repolarization pattern (J wave) and type 1 pattern in the inferior lead are considered predictors of cardiac events, it is possible that the deep and broad S wave that follows the R wave masks their presence and partially appears as R' [2, 3, 10, 27, 28]. Furthermore, considering that R' in lead II and aVF were not predictors, lead III may more selectively reflect abnormal right ventricular inferior/lateral wall potentials, which are important in BrS, given its vector direction. Indeed, some reports suggested the relationship between right ventricular volume overload and R' in inferior leads, as well as specific findings of the right coronary artery occlusion and lead III [29, 30].

### 4.2 Difference Between R' in Lead III and QRS Fragmentation

Morita et al. reported fragmented QRS in the inferior leads as a predictor of lethal events [11]. They defined it as three or more upward spikes in at least two consecutive leads; however, distinguishing small spikes from noise is sometimes challenging. The filter settings also influence the presence or absence of spikes. In this study, R' in lead III was determined based on a single lead only. The upward R wave was followed by a downward S wave that crossed the baseline once, and the upward excitation that followed the S wave and crossed the baseline again was defined as R', which is highly objective and clear. Furthermore, determining positivity based on only two upward excitations differs significantly from fragmented QRS in the inferior leads. These findings support its recognition as a new risk factor.

### 4.3 Previously Reported Predictors and Automated Analysis

This study did not find the QT/QTc interval, PQ interval, QRS duration, S‐wave in lead I, or R‐wave in lead aVR to be prognostic factors; although it is possible that these results could be reversed in a larger sample size [2, 6, 7, 8, 9, 12, 15, 16]. One possible reason for concern is that the automated analysis may have inaccurately measured the duration and amplitude. However, manual measurement, especially for low‐amplitude waves or those with difficult‐to‐determine inflection points, is generally challenging and may lack objectivity, raising concerns about its accuracy. Specialists can provide valid measurements; however, it is also important to establish a screening program that is universal and easy for non‐specialists to use. Fragmented QRS in the right precordial leads with ECG has also been reported as a predictive parameter of cardiac events [31]. This could not be analyzed using the automatic analysis software employed in this study. For fragmented QRS, the main concern is that the low‐pass filter setting and noise contamination have significant impacts on its determination. It is anticipated that automated analysis will enable accurate assessment of the fragmented QRS in the future.

### 4.4 Limitations

There are concerns regarding whether the automated assessment software accurately measures true values. At present, the electrocardiograph and its automatic analysis software used in this study are available only in Japan, and their usefulness should be confirmed in other countries. No validation cohort has been included in the analysis. ECG variability in BrS is a key issue, and future validation using repeated recordings, larger sample sizes, and data from multiple institutions is necessary. Integrated analysis of multiple ECG recordings may further improve the sensitivity and specificity of risk stratification.

## 5 Conclusions

Automated ECG analysis revealed that not only maximum cTpe in the right precordial leads but also R' duration ≥ 18 ms in lead III were predictors of VF/SCA in patients with BrS with spontaneous type 1 ECG pattern. Assessment using visual inspection also identified the presence of the R' wave in lead III as a novel independent predictor of VF/SCA. Although the study involved a small number of participants, epicardial mapping suggested that the fragmented potentials in the inferior/inferolateral wall of the right ventricle might reflect the R' wave in lead III, indicating the presence of the extensive arrhythmic substrate.

## Ethics Statement

This study adhered to institutional ethics and integrity guidelines. This study was approved by the Institutional Research Board of the National Cerebral and Cardiovascular Center (M22‐031‐13 and M26‐148‐16). The need for informed consent was waived by disclosing information about the study to the public and ensuring that study subjects had an opportunity to refuse to participate.

## Conflicts of Interest

Koji Miyamoto is affiliated with departments endowed with Medtronic. Kengo Kusano reports funding and grants received from Medtronic, Biosense Webster, and Abbott; and speakers' bureaus from Medtronic and Biosense Webster. However, none of these disclosures was directly associated with this study. The other authors declare no conflicts of interest.

## Supporting information


**Data S1:** Supporting information.

## Data Availability

The data supporting the findings of this study are available from the corresponding author upon reasonable request.
