# Supplementary material for: Automated Assessment to Predict Lethal Arrhythmias in Brugada Syndrome: Significance of R' in Lead III
Source: J Arrhythm. 2025 Aug 25;41(4):e70166. doi: 10.1002/joa3.70166 (PMC12375977; doi:10.1002/joa3.70166)
Supplement: Supplementary file 1 — Data S1: Supporting information. [file JOA3-41-e70166-s001.docx]

**Supplementary Table 1: Clinical parameters and follow-up events**

| **Characteristic** | **VF/SCA (+)**  N=28 | **VF/SCA (-)**  N=242 | **P value** |
| --- | --- | --- | --- |
| Age, years | 44±13 | 45±13 | 0.752 |
| Male, sex | 27 (98%) | 231 (96%) | 1.000 |
| FH of SCD | 5/26 (19%) | 51/171 (30%) | 0.807 |
| Induced VF with PES | 2/16 (13%) | 18/73 (25%) | 0.508 |
| *SCN5A* variant | 5/18 (28%) | 13/58 (22%) | 0.752 |
| ICD implantation | 25/28 (89%) | 48/194 (20%) | <0.001 |
| History of syncope | 12/28 (43%) | 45/197 (23%) | 0.006 |
| History of VF | 11/28 (39%) | 7/235 (3%) | <0.001 |

FH of SCD=family history of sudden cardiac death; ICD=implantable cardioverter defibrillator; PES=programmed electrical stimulation; SCA=sudden cardiac arrest; VF=ventricular fibrillation

**Supplementary Table 2: Electrocardiogram parameters and follow-up events**

|  | **VF/SCA (+)** | | **VF/SCA (-)** | |  |
| --- | --- | --- | --- | --- | --- |
|  | **median** | **First quartile-third quartile** | **median** | **First quartile-third quartile** | **P　value** |
| RR interval (ms) | 932 | 833-985 | 928 | 834-1028 | 0.740 |
| PR duration　(Ⅰ) (ms) | 177 | 162-196 | 170 | 156-190 | 0.246 |
| PR duration　(Ⅱ) (ms) | 190 | 170-201 | 176 | 162-194 | 0.081 |
| PR duration　(Ⅲ) (ms) | 184 | 170-201 | 172 | 158-192 | 0.044 |
| PR duration　(aVR) (ms) | 187 | 170-204 | 174 | 162-192 | 0.079 |
| PR duration　(aVL) (ms) | 178 | 168-192 | 168 | 154-190 | 0.053 |
| PR duration　(aVF) (ms) | 190 | 174-202 | 174 | 161-194 | 0.024 |
| PR duration　(V1) (ms) | 179 | 159-192 | 166 | 154-186 | 0.206 |
| PR duration　(V2) (ms) | 178 | 162-198 | 166 | 154-184 | 0.093 |
| PR duration　(V3) (ms) | 182 | 166-197 | 166 | 154-186 | 0.055 |
| PR duration　(V4) (ms) | 185 | 164-201 | 168 | 156-188 | 0.059 |
| PR duration　(V5) (ms) | 180 | 165-197 | 168 | 154-184 | 0.083 |
| PR duration　(V6) (ms) | 176 | 160-199 | 166 | 152-184 | 0.064 |
| QRS duration　(Ⅰ) (ms) | 112 | 102-127 | 104 | 94-114 | 0.046 |
| QRS duration　(Ⅱ) (ms) | 111 | 103-127 | 110 | 102-120 | 0.742 |
| QRS duration　(Ⅲ) (ms) | 114 | 106-129 | 112 | 104-122 | 0.568 |
| QRS duration　(aVR) (ms) | 106 | 98-122 | 106 | 96-112 | 0.318 |
| QRS duration　(aVL) (ms) | 109 | 102-124 | 108 | 98-118 | 0.375 |
| QRS duration　(aVF) (ms) | 114 | 110-125 | 112 | 104-122 | 0.897 |
| QRS duration　(V1) (ms) | 119 | 118-136 | 122 | 112-136 | 0.733 |
| QRS duration　(V2) (ms) | 126 | 120-147 | 122 | 114-136 | 0.061 |
| QRS duration　(V3) (ms) | 121 | 114-143 | 122 | 114-132 | 0.669 |
| QRS duration　(V4) (ms) | 117 | 110-138 | 114 | 108-126 | 0.201 |
| QRS duration　(V5) (ms) | 114 | 106-132 | 110 | 100-120 | 0.036 |
| QRS duration　(V6) (ms) | 110 | 102-127 | 107 | 98-118 | 0.100 |
| QRS duration　(V1; upper 1ICS) (ms) | 125 | 112-148 | 123 | 112-144 | 0.640 |
| QRS duration　(V2; upper 1ICS) (ms) | 118 | 111-148 | 118 | 110-140 | 0.839 |
| QRS duration　(V3; upper 1ICS) (ms) | 121 | 114-144 | 122 | 112-136 | 0.951 |
| QRS duration　(V1; upper 2ICS) (ms) | 130 | 116-140 | 126 | 114-140 | 0.702 |
| QRS duration　(V2; upper 2ICS) (ms) | 124 | 109-150 | 124 | 108-140 | 0.752 |
| QRS duration　(V3; upper 2ICS) (ms) | 117 | 112-144 | 120 | 110-136 | 0.851 |
| QT duration　(Ⅰ) (ms) | 376 | 360-405 | 378 | 358-392 | 0.638 |
| QT duration　(Ⅱ) (ms) | 404 | 380-414 | 393 | 378-412 | 0.575 |
| QT duration　(Ⅲ) (ms) | 385 | 372-411 | 389 | 370-410 | 0.822 |
| QT duration　(aVR) (ms) | 393 | 370-403 | 386 | 370-404 | 0.518 |
| QT duration　(aVL) (ms) | 378 | 358-391 | 372 | 354-390 | 0.336 |
| QT duration　(aVF) (ms) | 395 | 371-413 | 390 | 376-410 | 0.935 |
| QT duration　(V1) (ms) | 383 | 360-409 | 376 | 356-398 | 0.273 |
| QT duration　(V2) (ms) | 396 | 364-418 | 390 | 369-410 | 0.469 |
| QT duration　(V3) (ms) | 406 | 395-423 | 396 | 376-416 | 0.043 |
| QT duration　(V4) (ms) | 403 | 380-419 | 400 | 382-418 | 0.840 |
| QT duration　(V5) (ms) | 401 | 384-423 | 396 | 376-412 | 0.188 |
| QT duration　(V6) (ms) | 395 | 380-404 | 392 | 374-410 | 0.538 |
| QT duration　(V1; upper 1ICS) (ms) | 401 | 382-421 | 396 | 380-418 | 0.490 |
| QT duration　(V2; upper 1ICS) (ms) | 407 | 381-431 | 395 | 379-418 | 0.413 |
| QT duration　(V3; upper 1ICS) (ms) | 394 | 375-418 | 394 | 370-416 | 0.790 |
| QT duration　(V1; upper 2ICS) (ms) | 402 | 390-425 | 402 | 384-420 | 0.399 |
| QT duration　(V2; upper 2ICS) (ms) | 410 | 386-428 | 404 | 384-424 | 0.269 |
| QT duration　(V3; upper 2ICS) (ms) | 400 | 368-424 | 391 | 369-414 | 0.605 |
| QT max (V1-3; Normal-upper 2ICS) (ms) | 425 | 410-441 | 415 | 396-434 | 0.091 |
| QT max (all leads) (ms) | 430 | 413-441 | 416 | 396-434 | 0.086 |
| QTc duration　(Ⅰ) (ms) | 399 | 381-412 | 390 | 374-406 | 0.207 |
| QTc duration　(Ⅱ) (ms) | 419 | 396-433 | 409 | 397-426 | 0.388 |
| QTc duration　(Ⅲ) (ms) | 407 | 391-428 | 404 | 390-421 | 0.603 |
| QTc duration　(aVR) (ms) | 407 | 392-428 | 400 | 387-417 | 0.273 |
| QTc duration　(aVL) (ms) | 393 | 376-408 | 388 | 367-403 | 0.167 |
| QTc duration　(aVF) (ms) | 410 | 394-427 | 407 | 392-425 | 0.808 |
| QTc duration　(V1) (ms) | 402 | 370-426 | 393 | 373-412 | 0.230 |
| QTc duration　(V2) (ms) | 410 | 376-431 | 404 | 385-426 | 0.576 |
| QTc duration　(V3) (ms) | 428 | 403-443 | 412 | 396-426 | 0.024 |
| QTc duration　(V4) (ms) | 422 | 404-431 | 404 | 385-426 | 0.415 |
| QTc duration　(V5) (ms) | 421 | 401-444 | 411 | 396-426 | 0.024 |
| QTc duration　(V6) (ms) | 412 | 394-438 | 408 | 392-421 | 0.326 |
| QTc duration　(V1; upper 1ICS) (ms) | 417 | 397-446 | 411 | 393-432 | 0.267 |
| QTc duration　(V2; upper 1ICS) (ms) | 424 | 390-447 | 412 | 391-439 | 0.232 |
| QTc duration　(V3; upper 1ICS) (ms) | 409 | 395-430 | 408 | 387-429 | 0.428 |
| QTc duration　(V1; upper 2ICS) (ms) | 419 | 407-446 | 417 | 401-435 | 0.196 |
| QTc duration　(V2; upper 2ICS) (ms) | 424 | 411-461 | 421 | 399-439 | 0.146 |
| QTc duration　(V3; upper 2ICS) (ms) | 406 | 385-441 | 403 | 384-428 | 0.709 |
| QTc max (V1-3; Normal-upper 2ICS) (ms) | 441 | 425-487 | 431 | 415-451 | 0.048 |
| QTc max (all leads) (ms) | 444 | 425-487 | 433 | 416-452 | 0.053 |
| Tpeak-end　(Ⅰ) (ms) | 97 | 84-107 | 92 | 86-100 | 0.325 |
| Tpeak-end　(Ⅱ) (ms) | 104 | 98-110 | 105 | 99-109 | 0.877 |
| Tpeak-end　(Ⅲ) (ms) | 87 | 76-99 | 90 | 75-100 | 0.956 |
| Tpeak-end　(aVR) (ms) | 99 | 94-108 | 100 | 94-106 | 0.574 |
| Tpeak-end　(aVL) (ms) | 90 | 74-103 | 76 | 63-90 | 0.009 |
| Tpeak-end　(aVF) (ms) | 97 | 88-102 | 98 | 88-106 | 0.289 |
| Tpeak-end　(V1) (ms) | 96 | 74-114 | 88 | 72-105 | 0.435 |
| Tpeak-end　(V2) (ms) | 112 | 100-123 | 108 | 94-120 | 0.368 |
| Tpeak-end　(V3) (ms) | 122 | 110-130 | 118 | 108-124 | 0.069 |
| Tpeak-end　(V4) (ms) | 118 | 106-130 | 116 | 110-122 | 0.852 |
| Tpeak-end　(V5) (ms) | 112 | 106-118 | 108 | 104-116 | 0.239 |
| Tpeak-end　(V6) (ms) | 102 | 98-109 | 102 | 95-108 | 0.330 |
| Tpeak-end　(V1; upper 1ICS) (ms) | 100 | 72-106 | 90 | 80-102 | 0.325 |
| Tpeak-end　(V2; upper 1ICS) (ms) | 103 | 92-118 | 94 | 74-112 | 0.055 |
| Tpeak-end　(V3; upper 1ICS) (ms) | 118 | 103-123 | 110 | 98-120 | 0.084 |
| Tpeak-end　(V1; upper 2ICS) (ms) | 102 | 90-108 | 98 | 92-108 | 0.467 |
| Tpeak-end　(V2; upper 2ICS) (ms) | 96 | 86-119 | 96 | 80-108 | 0.323 |
| Tpeak-end　(V3; upper 2ICS) (ms) | 103 | 92-116 | 96 | 81-108 | 0.022 |
| Tpeak-end max (V1-3; Normal-upper 2ICS) | 134 | 122-146 | 124 | 116-134 | 0.013 |
| Tpeak-end max (all leads) (ms) | 135 | 122-146 | 124 | 118-136 | 0.015 |
| cTpeak-end　(Ⅰ) (ms) | 100 | 92-109 | 96 | 88-105 | 0.128 |
| cTpeak-end　(Ⅱ) (ms) | 109 | 103-113 | 107 | 101-114 | 0.681 |
| cTpeak-end　(Ⅲ) (ms) | 96 | 78-101 | 93 | 78-105 | 0.773 |
| cTpeak-end　(aVR) (ms) | 106 | 99-111 | 103 | 97-110 | 0.285 |
| cTpeak-end　(aVL) (ms) | 95 | 77-105 | 79 | 65-93 | 0.007 |
| cTpeak-end　(aVF) (ms) | 101 | 90-107 | 101 | 93-110 | 0.437 |
| cTpeak-end　(V1) (ms) | 98 | 76-132 | 93 | 72-108 | 0.456 |
| cTpeak-end　(V2) (ms) | 118 | 106-126 | 114 | 98-125 | 0.358 |
| cTpeak-end　(V3) (ms) | 127 | 116-138 | 121 | 113-130 | 0.172 |
| cTpeak-end　(V4) (ms) | 120 | 113-129 | 121 | 113-129 | 0.886 |
| cTpeak-end　(V5) (ms) | 115 | 112-129 | 113 | 107-121 | 0.193 |
| cTpeak-end　(V6) (ms) | 108 | 99-113 | 105 | 100-121 | 0.334 |
| cTpeak-end　(V1; upper 1ICS) (ms) | 105 | 73-118 | 93 | 84-117 | 0.195 |
| cTpeak-end　(V2; upper 1ICS) (ms) | 107 | 98-127 | 98 | 78-117 | 0.041 |
| cTpeak-end　(V3; upper 1ICS) (ms) | 120 | 109-136 | 113 | 101-125 | 0.061 |
| cTpeak-end　(V1; upper 2ICS) (ms) | 108 | 91-118 | 102 | 94-112 | 0.305 |
| cTpeak-end　(V2; upper 2ICS) (ms) | 107 | 95-126 | 100 | 83-115 | 0.196 |
| cTpeak-end　(V3; upper 2ICS) (ms) | 107 | 95-126 | 100 | 83-115 | 0.016 |
| cTpeak-end max (V1-3; Normal-upper 2ICS) | 141 | 132-148 | 129 | 120-141 | 0.002 |
| cTpeak-end max (all leads) (ms) | 142 | 132-152 | 131 | 122-142 | 0.002 |
| R wave amplitude (Ⅰ) (mV) | 0.58 | 0.43-0.64 | 0.50 | 0.34-0.66 | 0.247 |
| R wave amplitude (Ⅱ) (mV) | 0.80 | 0.55-1.03 | 0.94 | 0.68-1.24 | 0.058 |
| R wave amplitude (Ⅲ) (mV) | 0.32 | 0.12-0.62 | 0.54 | 0.23-0.80 | 0.119 |
| R wave amplitude (aVR) (mV) | 0.11 | 0.04-0.20 | 0.11 | 0.03-0.22 | 0.820 |
| R wave amplitude (aVL) (mV) | 0.24 | 0.16-0.44 | 0.18 | 0.08-0.35 | 0.086 |
| R wave amplitude (aVF) (mV) | 0.56 | 0.28-0.85 | 0.73 | 0.43-1.04 | 0.046 |
| R wave amplitude (V1) (mV) | 0.21 | 0.13-0.31 | 0.26 | 0.16-0.39 | 0.149 |
| R wave amplitude (V2) (mV) | 0.46 | 0.27-0.63 | 0.53 | 0.32-0.75 | 0.194 |
| R wave amplitude (V3) (mV) | 0.78 | 0.64-1.01 | 0.79 | 0.55-1.08 | 0.720 |
| R wave amplitude (V4) (mV) | 1.40 | 0.99-1.61 | 1.39 | 1.04-1.88 | 0.777 |
| R wave amplitude (V5) (mV) | 1.48 | 1.26-1.89 | 1.63 | 1.28-2.01 | 0.483 |
| R wave amplitude (V6) (mV) | 1.05 | 0.76-1.29 | 1.16 | 0.87-1.45 | 0.141 |
| R wave amplitude (V1; upper 1ICS) (mV) | 0.15 | 0.09-0.22 | 0.21 | 0.12-0.32 | 0.034 |
| R wave amplitude (V2; upper 1ICS) (mV) | 0.30 | 0.20-0,46 | 0.38 | 0.24-0.54 | 0.182 |
| R wave amplitude (V3; upper 1ICS) (mV) | 0.59 | 0.48-0.89 | 0.62 | 0.42-0.82 | 0.765 |
| R wave amplitude (V1; upper 2ICS) (mV) | 0.08 | 0.04-0.14 | 0.13 | 0.08-0.26 | 0.002 |
| R wave amplitude (V2; upper 2ICS) (mV) | 0.18 | 0.13-0,24 | 0.24 | 0.14-0.38 | 0.029 |
| R wave amplitude (V3; upper 2ICS) (mV) | 0.39 | 0.29-0.51 | 0.43 | 0.27-0.61 | 0.324 |
| R wave duration (Ⅰ) (ms) | 51 | 44-62 | 50 | 42-60 | 0.605 |
| R wave duration (Ⅱ) (ms) | 54 | 48-58 | 52 | 46-60 | 0.540 |
| R wave duration (Ⅲ) (ms) | 47 | 24-59 | 46 | 32-56 | 0.892 |
| R wave duration (aVR) (ms) | 31 | 16-48 | 30 | 18-48 | 0.847 |
| R wave duration (aVL) (ms) | 42 | 32-51 | 36 | 24-52 | 0.191 |
| R wave duration (aVF) (ms) | 56 | 46-65 | 50 | 44-60 | 0.182 |
| R wave duration (V1) (ms) | 32 | 28-41 | 34 | 30-40 | 0.720 |
| R wave duration (V2) (ms) | 39 | 34-46 | 40 | 34-44 | 0.792 |
| R wave duration (V3) (ms) | 46 | 42-50 | 44 | 38-52 | 0.552 |
| R wave duration (V4) (ms) | 50 | 46-56 | 54 | 50-58 | 0.033 |
| R wave duration (V5) (ms) | 48 | 44-55 | 48 | 42-56 | 0.687 |
| R wave duration (V6) (ms) | 50 | 42-56 | 48 | 42-54 | 0.361 |
| R wave duration (V1; upper 1ICS) (ms) | 29 | 28-37 | 32 | 28-38 | 0.275 |
| R wave duration (V2; upper 1ICS) (ms) | 36 | 32-46 | 36 | 32-42 | 0.737 |
| R wave duration (V3; upper 1ICS) (ms) | 44 | 40-50 | 42 | 36-48 | 0.212 |
| R wave duration (V1; upper 2ICS) (ms) | 25 | 23-30 | 30 | 24-34 | 0.022 |
| R wave duration (V2; upper 2ICS) (ms) | 32 | 28-38 | 34 | 28-38 | 0.377 |
| R wave duration (V3; upper 2ICS) (ms) | 41 | 36-47 | 48 | 34-44 | 0.447 |
| R’ wave amplitude (Ⅰ) (mV) | 0 | 0-0 | 0 | 0-0 | 0.742 |
| R’ wave amplitude (Ⅱ) (mV) | 0 | 0-0 | 0 | 0-0 | 0.105 |
| R’ wave amplitude (Ⅲ) (mV) | 0 | 0-0.1 | 0 | 0-0 | 0.032 |
| R’ wave amplitude (aVR) (mV) | 0 | 0-0 | 0 | 0-0 | 0.873 |
| R’ wave amplitude (aVL) (mV) | 0 | 0-0 | 0 | 0-0.08 | 0.053 |
| R’ wave amplitude (aVF) (mV) | 0 | 0-0 | 0 | 0-0 | 0.514 |
| R’ wave amplitude (V1) (mV) | 0 | 0-0.37 | 0 | 0-0.24 | 0.030 |
| R’ wave amplitude (V2) (mV) | 0 | 0-0.29 | 0.14 | 0-0.42 | 0.119 |
| R’ wave amplitude (V3) (mV) | 0 | 0-0 | 0 | 0-0 | 0.670 |
| R’ wave amplitude (V4) (mV) | 0 | 0-0 | 0 | 0-0 | 0.559 |
| R’ wave amplitude (V5) (mV) | 0 | 0-0 | 0 | 0-0 | 0.743 |
| R’ wave amplitude (V6) (mV) | 0 | 0-0 | 0 | 0-0 | NaN |
| R’ wave amplitude (V1; upper 1ICS) (mV) | 0.18 | 0-0.27 | 0.28 | 0.13-0.41 | 0.037 |
| R’ wave amplitude (V2; upper 1ICS) (mV) | 0.34 | 0-0.49 | 0.47 | 0.31-0.63 | 0.004 |
| R’ wave amplitude (V3; upper 1ICS) (mV) | 0 | 0-0 | 0 | 0-0.29 | 0.024 |
| R’ wave amplitude (V1; upper 2ICS) (mV) | 0.23 | 0.10-0.36 | 0.29 | 0.16-0.42 | 0.175 |
| R’ wave amplitude (V2; upper 2ICS) (mV) | 0.30 | 0-0.54 | 0.46 | 0.30-0.65 | 0.028 |
| R’ wave amplitude (V3; upper 2ICS) (mV) | 0.10 | 0-0.39 | 0.30 | 0-0,43 | 0.072 |
| R’ wave duration (Ⅰ) (ms) | 0 | 0-0 | 0 | 0-0 | 0.749 |
| R’ wave duration (Ⅱ) (ms) | 0 | 0-0 | 0 | 0-0 | 0.101 |
| R’ wave duration (Ⅲ) (ms) | 0 | 0-21 | 0 | 0-0 | 0.005 |
| R’ wave duration (aVR) (ms) | 0 | 0-0 | 0 | 0-0 | 0.872 |
| R’ wave duration (aVL) (ms) | 0 | 0-0 | 0 | 0-32 | 0.040 |
| R’ wave duration (aVF) (ms) | 0 | 0-0 | 0 | 0-0 | 0.529 |
| R’ wave duration (V1) (ms) | 0 | 0-0 | 0 | 0-44 | 0.039 |
| R’ wave duration (V2) (ms) | 0 | 0-0 | 14 | 0-42 | 0.396 |
| R’ wave duration (V3) (ms) | 0 | 0-0 | 0 | 0-0 | 0.678 |
| R’ wave duration (V4) (ms) | 0 | 0-0 | 0 | 0-0 | 0.559 |
| R’ wave duration (V5) (ms) | 0 | 0-0 | 0 | 0-0 | 0.743 |
| R’ wave duration (V6) (ms) | 0 | 0-0 | 0 | 0-0 | NaN |
| R’ wave duration (V1; upper 1ICS) (ms) | 38 | 0-69 | 44 | 22-72 | 0.295 |
| R’ wave duration (V2; upper 1ICS) (ms) | 30 | 0-49 | 42 | 30-66 | 0.007 |
| R’ wave duration (V3; upper 1ICS) (ms) | 0 | 0-0 | 0 | 0-42 | 0.025 |
| R’ wave duration (V1; upper 2ICS) (ms) | 45 | 25-67 | 56 | 34-72 | 0.439 |
| R’ wave duration (V2; upper 2ICS) (ms) | 45 | 0-69 | 52 | 32-74 | 0.499 |
| R’ wave duration (V3; upper 2ICS) (ms) | 13 | 0-54 | 37 | 0-56 | 0.155 |
| S wave amplitude (Ⅰ) (mV) | -0.19 | -0.25 - -0.07 | -0.09 | -0.21 - -0.01 | 0.069 |
| S wave amplitude (Ⅱ) (mV) | -0.19 | -0.28 - -0.08 | -0.19 | -0.34 - -0.09 | 0.442 |
| S wave amplitude (Ⅲ) (mV) | -0.17 | -0.31 - -0.08 | -0.17 | -0.30 - -0.08 | 0.888 |
| S wave amplitude (aVR) (mV) | 0 | -0.08 - 0 | 0 | -0.55 - 0 | 0.534 |
| S wave amplitude (aVL) (mV) | -0.17 | -0.25 - 0 | -0.12 | -0.31 - 0 | 0.916 |
| S wave amplitude (aVF) (mV) | -0.13 | -0.24 - -0.04 | -0.17 | -0.31 - -0.07 | 0.232 |
| S wave amplitude (V1) (mV) | -0.80 | -1.18 - -0.50 | -0.81 | -1.09 - -0.53 | 0.781 |
| S wave amplitude (V2) (mV) | -1.08 | -1.54 – -0.73 | -1.10 | -1.57 - -0.70 | 0.743 |
| S wave amplitude (V3) (mV) | -1.19 | -1.67 – 0.77 | -1.12 | -1.52 – -0.86 | 0.896 |
| S wave amplitude (V4) (mV) | -0.78 | -1.10 - -0.67 | -1.12 | -1.52 - -0.86 | 0.220 |
| S wave amplitude (V5) (mV) | -0.45 | -0.57 - -0.28 | -0.35 | -0.53 - -0.18 | 0.154 |
| S wave amplitude (V6) (mV) | -0.21 | -0.27 - -0.10 | -0.16 | -0.26 - -0.06 | 0.438 |
| S wave amplitude (V1; upper 1ICS) (mV) | -0.73 | -1.02 - -0.47 | -0.71 | -0.96 - -0.47 | 0.839 |
| S wave amplitude (V2; upper 1ICS) (mV) | -0.83 | -1.22 - -0.46 | -0.86 | -1.21 - -0.48 | 0.845 |
| S wave amplitude (V3; upper 1ICS) (mV) | -0.96 | -1.42 - -0.61 | -0.94 | -1.37 - -0.66 | 0.953 |
| S wave amplitude (V1; upper 2ICS) (mV) | -0.65 | -0.88 - -0.38 | -0.74 | -0.95 - -0.46 | 0.360 |
| S wave amplitude (V2; upper 2ICS) (mV) | -0.69 | -0.96 - -0.43 | -0.76 | -1.00 - -0.44 | 0.492 |
| S wave amplitude (V3; upper 2ICS) (mV) | -0.72 | -1.11 - -0.42 | -0.79 | -1.10 - -0.45 | 0.569 |
| S wave duration (Ⅰ) (ms) | 40 | 22-54 | 28 | 10-42 | 0.068 |
| S wave duration (Ⅱ) (ms) | 41 | 22-57 | 44 | 26-56 | 0.856 |
| S wave duration (Ⅲ) (ms) | 32 | 14-51 | 40 | 14-62 | 0.159 |
| S wave duration (aVR) (ms) | 0 | 0-0 | 0 | 0-38 | 0.619 |
| S wave duration (aVL) (ms) | 39 | 0-62 | 26 | 0-42 | 0.133 |
| S wave duration (aVF) (ms) | 36 | 9-56 | 42 | 22-58 | 0.308 |
| S wave duration (V1) (ms) | 51 | 40-61 | 48 | 36-56 | 0.288 |
| S wave duration (V2) (ms) | 48 | 40-55 | 44 | 34-54 | 0.226 |
| S wave duration (V3) (ms) | 52 | 42-62 | 46 | 40-56 | 0.133 |
| S wave duration (V4) (ms) | 52 | 45-66 | 42 | 32-52 | <0.001 |
| S wave duration (V5) (ms) | 56 | 45-66 | 44 | 34-54 | 0.001 |
| S wave duration (V6) (ms) | 48 | 38-63 | 42 | 29-54 | 0.052 |
| S wave duration (V1; upper 1ICS) (ms) | 41 | 34-55 | 38 | 30-46 | 0.025 |
| S wave duration (V2; upper 1ICS) (ms) | 37 | 29-51 | 34 | 26-42 | 0.159 |
| S wave duration (V3; upper 1ICS) (ms) | 46 | 34-53 | 40 | 34-50 | 0.310 |
| S wave duration (V1; upper 2ICS) (ms) | 38 | 30-47 | 36 | 28-42 | 0.210 |
| S wave duration (V2; upper 2ICS) (ms) | 36 | 26-44 | 32 | 26-38 | 0.126 |
| S wave duration (V3; upper 2ICS) (ms) | 40 | 28-47 | 36 | 28-44 | 0.309 |
| J wave amplitude (Ⅰ) (mV) | 0 | 0-0 | 0 | 0-0 | 0.054 |
| J wave amplitude (Ⅱ) (mV) | 0 | 0-0 | 0 | 0-0 | 0.050 |
| J wave amplitude (Ⅲ) (mV) | 0 | 0-0.02 | 0 | 0-0 | 0.559 |
| J wave amplitude (aVR) (mV) | 0 | 0-0 | 0 | 0-0 | NaN |
| J wave amplitude (aVL) (mV) | 0 | 0-0 | 0 | 0-0 | 0.403 |
| J wave amplitude (aVF) (mV) | 0 | 0-0 | 0 | 0-0 | 0.297 |
| J wave amplitude (V1) (mV) | 0 | 0-0 | 0 | 0-0 | NaN |
| J wave amplitude (V2) (mV) | 0 | 0-0 | 0 | 0-0 | NaN |
| J wave amplitude (V3) (mV) | 0 | 0-0 | 0 | 0-0 | NaN |
| J wave amplitude (V4) (mV) | 0 | 0-0 | 0 | 0-0 | 0.365 |
| J wave amplitude (V5) (mV) | 0 | 0-0 | 0 | 0-0 | 0.302 |
| J wave amplitude (V6) (mV) | 0 | 0-0 | 0 | 0-0 | 0.743 |
| J wave amplitude (V1; upper 1ICS) (mV) | 0 | 0-0 | 0 | 0-0 | NaN |
| J wave amplitude (V2; upper 1ICS) (mV) | 0 | 0-0 | 0 | 0-0 | NaN |
| J wave amplitude (V3; upper 1ICS) (mV) | 0 | 0-0 | 0 | 0-0 | NaN |
| J wave amplitude (V1; upper 2ICS) (mV) | 0 | 0-0 | 0 | 0-0 | NaN |
| J wave amplitude (V2; upper 2ICS) (mV) | 0 | 0-0 | 0 | 0-0 | NaN |
| J wave amplitude (V3; upper 2ICS) (mV) | 0 | 0-0 | 0 | 0-0 | NaN |
| J wave duration (Ⅰ) (ms) | 0 | 0-0 | 0 | 0-0 | 0.054 |
| J wave duration (Ⅱ) (ms) | 0 | 0-0 | 0 | 0-0 | 0.058 |
| J wave duration (Ⅲ) (ms) | 0 | 0-0 | 0 | 0-0 | 0.559 |
| J wave duration (aVR) (ms) | 0 | 0-0 | 0 | 0-0 | NaN |
| J wave duration (aVL) (ms) | 0 | 0-0 | 0 | 0-0 | 0.403 |
| J wave duration (aVF) (ms) | 0 | 0-0 | 0 | 0-0 | 0.304 |
| J wave duration (V1) (ms) | 0 | 0-0 | 0 | 0-0 | NaN |
| J wave duration (V2) (ms) | 0 | 0-0 | 0 | 0-0 | NaN |
| J wave duration (V3) (ms) | 0 | 0-0 | 0 | 0-0 | NaN |
| J wave duration (V4) (ms) | 0 | 0-0 | 0 | 0-0 | 0.365 |
| J wave duration (V5) (ms) | 0 | 0-0 | 0 | 0-0 | 0.302 |
| J wave duration (V6) (ms) | 0 | 0-0 | 0 | 0-0 | 0.743 |
| J wave duration (V1; upper 1ICS) (ms) | 0 | 0-0 | 0 | 0-0 | NaN |
| J wave duration (V2; upper 1ICS) (ms) | 0 | 0-0 | 0 | 0-0 | NaN |
| J wave duration (V3; upper 1ICS) (ms) | 0 | 0-0 | 0 | 0-0 | NaN |
| J wave duration (V1; upper 2ICS) (ms) | 0 | 0-0 | 0 | 0-0 | NaN |
| J wave duration (V2; upper 2ICS) (ms) | 0 | 0-0 | 0 | 0-0 | NaN |
| J wave duration (V3; upper 2ICS) (ms) | 0 | 0-0 | 0 | 0-0 | NaN |

ICS=intercostal space; SCA=sudden cardiac arrest; VF=ventricular fibrillation

**Supplementary Table 3: Cut-off values and univariate data on event occurrence**

|  | **Cut-off value**  **(sensitivity: specificity)** | **AUC** | **95% CI (cut-off value)** | **HR** | **95% CI (event)** | **P value** |
| --- | --- | --- | --- | --- | --- | --- |
| PR duration (Ⅲ) | 180(0.64:0.61) | 0.62 | 0.50–0.73 | 2.33 | 1.09–4.98 | 0.029 |
| PR duration (aVF) | 180(0.65:0.64) | 0.63 | 0.52–0.74 | 2.37 | 1.10–5.15 | 0.028 |
| QRS duration (Ⅰ) | 106(0.55:0.71) | 0.62 | 0.50–0.74 | 2.46 | 1.08–5.61 | 0.032 |
| QRS duration (V5) | 130(0.88:0.32) | 0.62 | 0.52–0.73 | 2.46 | 1.09–5.51 | 0.029 |
| QT duration (V3) | 404(0.59:0.64) | 0.62 | 0.52–0.72 | 2.42 | 1.11–5.27 | 0.026 |
| QTc duration (V3) | 430(0.79:0.50) | 0.63 | 0.51–0.75 | 3.12 | 1.48–6.55 | 0.003 |
| QTc max duration (V1-3; Normal-upper 2ICS) | 481(0.92:0.36) | 0.61 | 0.49–0.74 | 4.64 | 2.14–10.08 | <0.001 |
| Tp-e duration (aVL) | 98(0.86:0.43) | 0.65 | 0.54–0.76 | 4.17 | 1.97–8.84 | <0.001 |
| Tp-e duration (V3; upper 2ICS) | 90(0.37:0.82) | 0.63 | 0.53–0.74 | 1.16 | 0.16–8.53 | 0.890 |
| Tp-e max duration (V1-3; Normal-upper 2ICS) | 132(0.70:0.57) | 0.64 | 0.53–0.76 | 2.82 | 1.32–6.00 | 0.007 |
| Tp-e max duration (all leads) | 136(0.74,0.50) | 0.64 | 0.53–0.76 | 2.63 | 1.25–5.54 | 0.011 |
| cTp-e duration (aVL) | 93.02(0.75:0.57) | 0.66 | 0.55–0.76 | 3.80 | 1.79–8.03 | <0.001 |
| cTp-e duration (V2; upper 1ICS) | 90.36(0.41:0.86) | 0.62 | 0.51–0.73 | 3.50 | 1.21–10.1 | 0.020 |
| cTp-e duration (V3; upper 2ICS) | 87.61(0.31:0.96) | 0.64 | 0.54–0.74 | 9.64 | 1.31–70.9 | 0.026 |
| cTp-e max duration (V1-3; Normal-upper 2ICS) | 137.18(0.69:0.68) | 0.68 | 0.57–0.78 | 3.84 | 1.73–8.53 | <0.001 |
| cTp-e max duration (all leads) | 137.18(0.65:0.71) | 0.68 | 0.57–0.78 | 3.82 | 1.68–8.72 | <0.001 |
| R amplitude (aVF) | 0.59(0.62:0.64) | 0.62 | 0.50–0.73 | 0.45 | 0.21–0.97 | 0.043 |
| R amplitude (V1; upper 1ICS) | 0.18(0.56:0.68) | 0.62 | 0.52–0.72 | 0.49 | 0.22–1.06 | 0.070 |
| R amplitude (V1; upper 2ICS) | 0.16(0.42-0.86) | 0.68 | 0.57–0.78 | 0.33 | 0.13–0.87 | 0.025 |
| R amplitude (V2; upper 2ICS) | 0.26(0.44-0.86) | 0.63 | 0.52–0.73 | 0.39 | 0.16–0.96 | 0.040 |
| R duration (V4) | 48(0.76:0.43) | 0.62 | 0.51–0.73 | 0.37 | 0.17–0.81 | 0.014 |
| R duration (V1; upper 2ICS) | 28(0.69:0.50) | 0.56 | 0.45–0.68 | 0.45 | 0.21–0.96 | 0.040 |
| R' amplitude (Ⅲ) | 0.04(0.83:0.36) | 0.59 | 0.49–68 | 2.55 | 1.17–5.57 | 0.019 |
| R' amplitude (V1) | 0.54(0.94:0.11) | 0.39 | 0.30–0.48 | 2.09 | 0.63–6.94 | 0.230 |
| R' amplitude (V1; upper 1ICS) | 0.26(0.53:0.75) | 0.62 | 0.51–0.73 | 0.37 | 0.16–0.84 | 0.018 |
| R' amplitude (V2; upper 1ICS) | 0.41(0.61:0.71) | 0.67 | 0.56–0.78 | 0.32 | 0.14–0.71 | 0.005 |
| R' amplitude (V3; upper 1ICS) | 0.72(0.99:0.04) | 0.39 | 0.30–0.47 | 3.16 | 0.43–23.43 | 0.260 |
| R' amplitude (V2; upper 2ICS) | 0.32(0.74:0.57) | 0.63 | 0.51–0.74 | 0.37 | 0.18–0.78 | 0.009 |
| R' duration (Ⅲ) | 18(0.87:0.39) | 0.61 | 0.52–0.71 | 3.68 | 1.71–7.93 | <0.001 |
| R' duration (aVL) | 32(0.24:0.96) | 0.60 | 0.53–0.66 | 6.75 | 0.92–49.7 | 0.061 |
| R' duration (V1) | 0(0.43:0.82) | 0.61 | 0.51–0.70 | 3.17 | 1.20–8.34 | 0.020 |
| R' duration (V2; upper 1ICS) | 30(0.74:0.57) | 0.66 | 0.54–0.78 | 0.41 | 0.20–0.87 | 0.020 |
| R' duration (V3; upper 1ICS) | 86(0.97:0.04) | 0.39 | 0.30–0.47 | 1.28 | 0.17–9.43 | 0.810 |
| S duration (V4) | 46(0.58:0.75) | 0.70 | 0.60–0.80 | 3.27 | 1.39–7.71 | 0.007 |
| S duration (V5) | 50(0.63:0.68) | 0.69 | 0.59–0.79 | 2.63 | 1.19–5.84 | 0.017 |
| S duration (V1; upper 1ICS) | 54(0.89:0.36) | 0.63 | 0.51–0.75 | 3.25 | 1.48–7.15 | 0.003 |

AUC=area under the curve; CI=confidence interval; HR=hazard ratio; ICS=intercostal space; Tp-e=Tpeak-end; cTp-e=Tp-e intervals corrected for heart rate by Bazett's formula
